# CO_2_ activation by copper oxide clusters: size, composition, and charge state dependence†

**DOI:** 10.1039/d4cp02651a

**Published:** 2024-09-02

**Authors:** Pavol Mikolaj, Barbara Zamora Yusti, László Nyulászi, Joost M. Bakker, Tibor Höltzl, Sandra M. Lang

**Affiliations:** a Institute of Surface Chemistry and Catalysis, University of Ulm Ulm 89069 Germany sandra.lang@uni-ulm.de; b Department of Inorganic and Analytical Chemistry, Budapest University of Technology and Economics Műegytem rkp. 3 Budapest-1111 Hungary; c HUN-REN-BME Computation Driven Chemistry research group Műegytem rkp. 3 Budapest-1111 Hungary; d Radboud University, Institute of Molecules and Materials, FELIX Laboratory 6525 ED Nijmegen The Netherlands; e Furukawa Electric Institute of Technology, Nanomaterials Science Group Késmárk utca 28/A Budapest 1158 Hungary tibor.holtzl@furukawaelectric.com

## Abstract

The interaction of CO_2_ with copper oxide clusters of different size, composition, and charge is investigated *via* infrared multiple-photon dissociation (IR-MPD) spectroscopy and density functional theory (DFT) calculations. Laser ablation of a copper target in the presence of an O_2_/He mixture leads to the preferred formation of oxygen-rich copper oxide cluster cations, Cu_*x*_O_*y*_^+^ (*y* > *x*; *x* ≤ 8), while the anionic cluster distribution is dominated by stoichiometric (*x* = *y*) and oxygen-deficient (*y* < *x*; *x* ≤ 8) species. Subsequent reaction of the clusters with CO_2_ in a flow tube reactor results in the preferred formation of near-stoichiometric Cu_*x*_O_*y*_(CO_2_)^+/−^ complexes. IR-MPD spectroscopy of the formed complexes reveals the non-activated binding of CO_2_ to all cations while CO_2_ is activated by all anions. The great resemblance of spectra for all sizes investigated demonstrates that CO_2_ activation is largely independent of cluster size and Cu/O ratio but mainly determined by the cluster charge state. Comparison of the IR-MPD spectra with DFT calculations of the model systems Cu_2_O_4_(CO_2_)^−^ and Cu_3_O_4_(CO_2_)^−^ shows that CO_2_ activation exclusively results in the formation of a CO_3_ unit. Subsequent CO_2_ dissociation to CO appears to be unfavorable due to the instability of CO on the copper oxide clusters indicating that potential hydrogenation reactions will most likely proceed *via* formate or bicarbonate intermediates.

## Abstract 2

Infrared multiple-photon dissociation (IR-MPD) spectroscopy and density functional theory (DFT) calculations reveal a strongly charge dependent activation of CO_2_ by copper oxide clusters.

## Graphical abstract

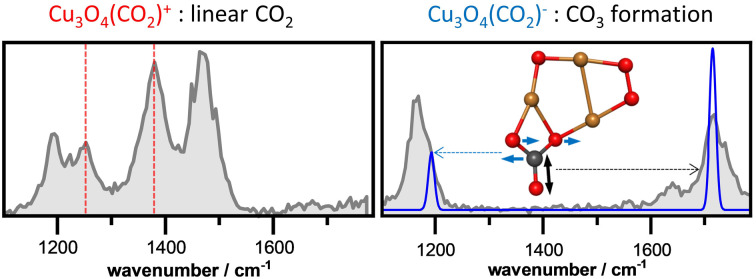

## 1 Introduction

Based on the possibility of harnessing renewable energy to form chemical products and to reduce the environmental impact of the current petrochemical-based industry, extensive research has accelerated the development of better catalysts for the efficient CO_2_ conversion into valuable products.^1^ Copper based catalysts are well known for their high activity.^2–4^ Besides copper, oxygen plays an important role in these catalysts, *e.g.*, at the copper metal-oxide interface in thermal catalysis^5,6^ or in electrocatalysis using oxide derived copper,^7–9^ where the subsurface oxygen helps to stabilize the activated (chemisorbed) bent CO_2_ on the catalyst surface^10^ and can enhance the formation of C_2+_ products. It is worth noting that copper-oxide itself is also catalytically active, *e.g.*, in oxidative dehydrogenation of alcohols,^11^ propylene partial oxidation,^12^ or in electrocatalysis for oxygen evolution reaction.^13^ Copper–oxygen intermediates often play an important role in different oxygenation and oxidation reactions, including biologically important cases.^14^

Nanoparticles or clusters are promising catalyst candidates on their own, as well as model compounds whose investigation provides invaluable information to understand the influence of the active site composition and charge in bulk catalysts.^15^ Accordingly, (sub-)nanometer copper oxide clusters have become popular in heterogeneous catalysis because of their catalytic activities in selective oxidation reactions, *e.g.*, in the reduction of NO and CO_2_,^16^ or the methane-to-methanol conversion.^17^ It has also been shown that copper oxide nanoparticles effectively catalyze the formation of environmentally hazardous molecules, such as polychlorinated dibenzo-*p*-dioxins and dibenzofurans.^18^ Amorphous Cu(ii) oxide nanoclusters are reported to be highly active for producing CO both in photo- and electrocatalysis.^19^ Alumina-supported Cu_4_ clusters were found to catalyze the CO_2_ hydrogenation to methanol with an exceptionally high turn-over frequency.^20^

Rooted in the central role that metal nanoparticles have in heterogeneous catalysis, fundamental research in physical chemistry has provided a set of tools to study simplified systems such as metal nanoparticles and an important subclass constituted by metal clusters. These model systems are characterized by their exact elementary composition and charge state.^21^ Isolated copper oxide clusters were investigated experimentally by negative ion photoelectron spectroscopy,^22^ ion-mobility^23^ and thermal desorption mass spectrometry.^24^ Quantum chemical calculations were carried out for certain charged and neutral copper oxide clusters.^23,25–29^

Gas-phase vibrational spectroscopy in conjunction with theoretical first-principles calculations is a useful tool to reveal the structure of metal clusters and their reaction products and thus provides insight into the binding and activation of small molecules.^15,30^ In the present work, we have employed these techniques to study the potential activation of CO_2_ by free copper oxide clusters as a function of charge state (+1, −1) as well as cluster size and composition (Cu_*x*_O_*y*_^+^ with *x* = 1–6 and Cu_*x*_O_*y*_^−^ with *x* = 1–3).

## 2 Methods

### 2.1 Experimental methods

Copper oxide clusters were produced *via* laser ablation of an isotopically enriched ^65^Cu foil in the presence of a 1% O_2_/He mixture and subsequently reacted with 1.7% CO_2_/He mixture in an adjacent flow tube reactor, which was held at room temperature throughout the experiments. The reaction mixture was then expanded into vacuum forming a molecular beam before entering the intracavity region where it was irradiated by the IR laser beam of the Free-Electron Laser for Intra Cavity Experiments (FELICE, 240–1800 cm^−1^; 10 μs pulse duration, spectral bandwidth 0.5% FWHM of the central wavenumber) crossing it at an angle of 35°. A few μs after the interaction with FELICE, all clusters were extracted into a reflectron time-of-flight mass spectrometer by a set of pulsed high voltage plates and detected with a microchannel plate detector.^31,32^

To correct for long-term source fluctuations, the experiment was operated at twice the FELICE repetition rate, allowing for the recording of reference mass spectra in between successive FELICE pulses. Whenever FELICE was in resonance with an IR active vibrational mode of a given cluster, multiple IR photons were absorbed sequentially, leading to heating of the complex and finally to its fragmentation. The IR-MPD spectra shown in this contribution represent the depletion yield *Y*(*ν̃*) at wavenumber *ν̃*, calculated as *Y*(*ν̃*) = −ln[*I*(*ν̃*)/*I*_0_], where *I*(*ν̃*) and *I*_0_ are the mass peak intensities with and without laser light, respectively. To reduce the IR fluence with which the complexes are irradiated, the whole instrument can be translated up to 300 mm from the focus position leading to a 30-fold reduction in intensity but increased overlap between the laser and molecular beam and thus an increased signal to noise ratio. All spectra presented in this work were recorded between 300 and 290 mm from the focus. To further reduce the IR fluence and increase the spectral resolution, the overlap between laser and molecular beams can be purposely misaligned such that the molecular beam only observes the lower intensity part of the laser beam. Information on this position is given in the caption of Fig. 3 below.

For comparison between the experimental and theoretical spectra, the intensity of the latter were divided by the wavenumber, which we find leads to a better match of relative intensities. We rationalize this as due to the inverse proportionality of the laser's spectral brightness to wavenumber, mirroring the proportionality of the spectral bandwidth to wavenumber (*cf.* Section S1 of the ESI†).

### 2.2 Theoretical methods

The search for the global minimum bare cluster geometries of Cu_2_O_4_^−^ and Cu_3_O_4_^−^ (which were chosen as representatives for the experimentally investigated clusters) was performed using the CALYPSO program,^33^ a swarm-intelligence based structure prediction method, interfaced with the Gaussian 16^34^ package for local structure optimization in different spin states. For the structure relaxation, the geometry optimizations were carried out using the LANL2DZ basis set and BP86 functional without any symmetry constraint. Further optimization and analytical second derivatives of the molecular energy with respect to the nuclear coordinates were computed on the optimized structures obtained from CALYPSO to confirm that each located structure had no imaginary vibrational frequency, *i.e.* true minima are located. For the post-optimization of the CALYPSO found bare clusters, the def2-TZVPD basis set and the TPSSh functional were used, implemented in the Q-Chem^35^ software. While the polarized triple-*ζ* quality def2-TZVP basis set has been found to yield accurate CO_2_ binding energies for different (doped) copper clusters,^36,37^ here we also included diffuse functions to ensure the adequate description of the anion electronic structures.

To systematically investigate carbon dioxide adsorption in different binding modes to low-energy bare copper-oxide clusters, we used an in-house code as *e.g.* in ref. 36. In the binding modes considered, CO_2_ was either non-activated (in η_1_(O) binding mode), activated (in η_2_(C,O) binding mode; activation is evidenced from a bending of the CO_2_ moiety and an increased CO distance), or dissociated – decomposing into CO and O. The ESI,† specifically section S6, provides comprehensive data on the energies, spin multiplicities and XYZ coordinates.

## 3 Results and discussion

### 3.1 Formation and reactivity of copper oxide clusters

**Fig. 1:**
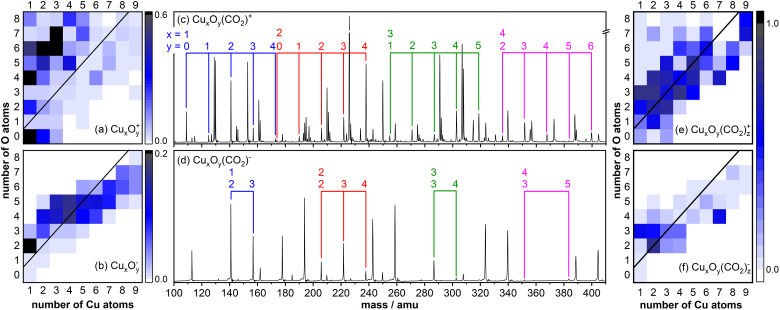
(a) and (b) Mass spectral intensities of the (a) cationic and (b) anionic copper oxide cluster distributions produced *via* laser ablation (intensities are given in arbitrary units). The black lines in (a) and (b) indicate stoichiometric clusters Cu_*x*_O_*y*_^+/−^ (*x* = *y*). (c) and (d) Mass spectra obtained after reaction of (c) cationic and (d) anionic clusters with CO_2_ in a flow tube reactor. The colored indicators identify the mass peaks of Cu_*x*_O_*y*_(CO_2_)^+/−^. (e) and (f) Adsorption efficiency for (e) cationic and (f) anionic clusters calculated with eqn (1).

Fig. 1 displays typical copper oxide cluster distributions for cationic (Fig. 1a) and anionic (Fig. 1b) species as color map representations. Under these cluster production conditions, Cu_*x*_O_*y*_^+^ cations with up to eight copper and nine oxygen atoms are formed and the distribution is dominated by highly oxidized (*y* > *x*) clusters. In contrast, the size distribution of anionic Cu_*x*_O_*y*_^−^ clusters is narrower, and the mass spectrum is dominated by stoichiometric (*x* = *y*) and slightly oxygen-rich clusters for *x* ≤ 4 as well as oxygen-deficient (*y* < *x*) clusters for *x* > 4. In addition, while Cu^+^ and CuO_*y*_^+^ (*y* = 2,4,6) are formed in considerable amounts, the anionic counterparts are hardly produced.

Subsequent reaction of the clusters with CO_2_ in a flow tube reactor typically leads to the adsorption of one or two CO_2_ molecules. Fig. 1c and d display mass spectra obtained for (c) cationic and (d) anionic clusters showing that Cu_*x*_O_*y*_(CO_2_)^+^, products are mainly formed for oxygen deficient and near stoichiometric cationic clusters, while products of highly oxidized clusters (*y* > *x* + 2) are not observed in notable quantities. This can be either caused by the inherent inactivity of these clusters towards CO_2_ or by reaction *via* O_2_/CO_2_ exchange (Cu_*x*_O_*y*_^+^ + CO_2_ → Cu_*x*_O_*y*−2_(CO_2_)^+^ + O_2_). In any case, this shows that Cu_*x*_O_*y*_(CO_2_)^+^ complexes with excess oxygen content are rather unstable. In case of anionic clusters only few Cu_*x*_O_*y*_(CO_2_)^−^ products with sufficient intensity are produced. This is on the one hand caused by the narrower size distribution of the bare Cu_*x*_O_*y*_^−^ clusters (Fig. 1b) but on the other hand the mass spectra also show that Cu_*x*_O_*y*_(CO_2_)^−^ products are mainly formed on a much smaller number of clusters, limited to near-stoichiometric clusters (with *x* ≤ 3 and *y* ≤ *x* + 1), while larger clusters (with *x* > 3) appear to be rather unreactive. This is also illustrated in a color map representation (Fig. 1e and f) showing the adsorption efficiency (AE) calculated as1
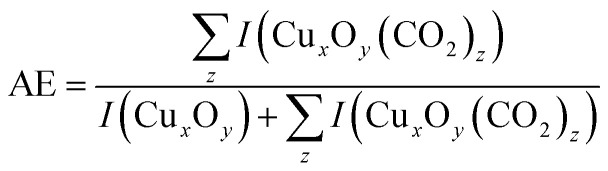
where *I* denotes the intensity of the mass peak as extracted from the mass spectrum.

### 3.2 IR-MPD spectra of cationic and anionic Cu_*x*_O_*y*_(CO_2_) complexes

Although mass spectrometry can provide information on the overall reactivity, it does not give insight into the potential activation of CO_2_ or bonding motifs. Therefore, we have additionally probed the formed Cu_*x*_O_*y*_(CO_2_) complexes *via* IR-MPD spectroscopy.

Free CO_2_ is linear and has four normal modes: the symmetric (*ν*_1_ = 1333 cm^−1^) and asymmetric (*ν*_3_ = 2349 cm^−1^) stretching vibration and the doubly degenerate bending vibration (*ν*_2_ = 667 cm^−1^).^38^*ν*_1_ and the first overtone of *ν*_2_ couple into a Fermi resonance and instead of a single peak at 1333 cm^−1^ typically a pair of peaks is observed at 1388 cm^−1^ and 1285 cm^−1^ (*ν*_+_ and *ν*_−_).^39–42^ Although IR-inactive for gas-phase CO_2_, the Fermi dyad can become IR-active due to symmetry breaking when CO_2_ is weakly adsorbed on extended metal oxide surfaces^43^ or clusters.^44,45^ In contrast, if CO_2_ is activated by partial electron transfer from the (cluster) surface, the C–O bonds weaken and the molecule adopts a bent geometry.^46,47^ This change of geometry results in the breakdown of the Fermi dyad and a red-shift of the asymmetric stretching mode – depending on the binding geometry – by several hundreds of wavenumbers.^30,48–50^ Thus, weak adsorption of CO_2_ on a (cluster) surface can be clearly distinguished from activated adsorption *via* infrared spectroscopy.

**Fig. 2:**
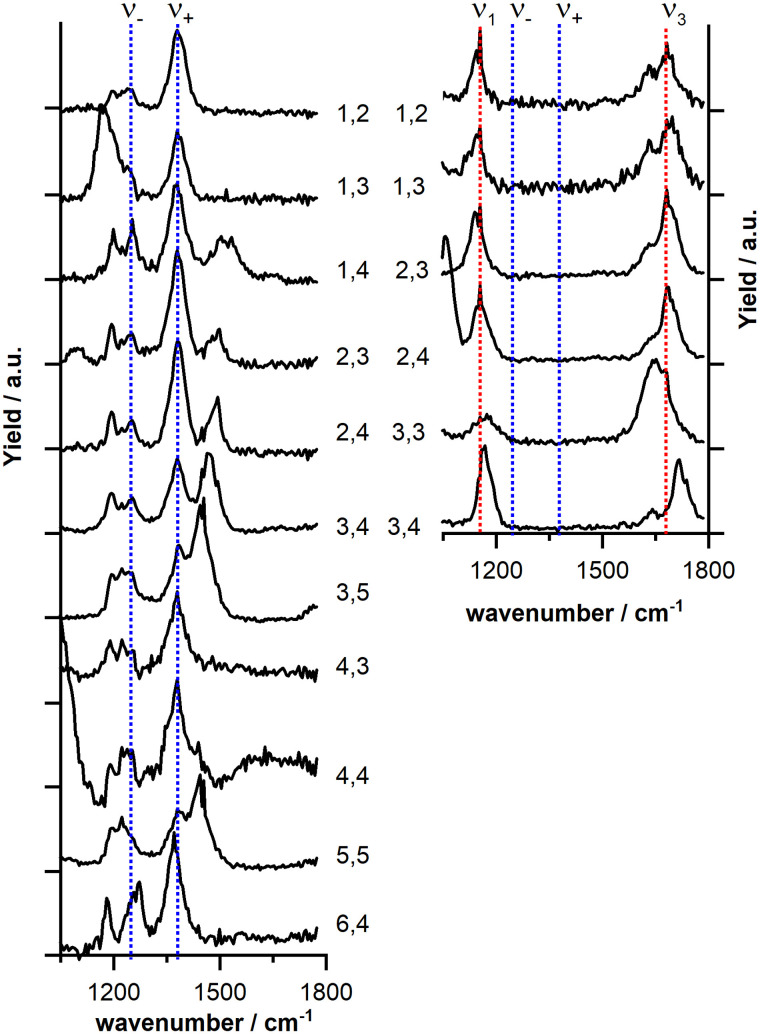
IR-MPD spectra of (left column) cationic Cu_*x*_O_*y*_(CO_2_)^+^ and (right column) anionic Cu_*x*_O_*y*_(CO_2_)^−^ complexes (labeled *x*,*y*). The blue dotted lines indicate the Fermi dyad and the red dotted lines modes of activated CO_2_. The black tick marks on the *y*-axis indicate the zero depletion yield for each spectrum. The spectra of the anionic complexes were recorded at reduced IR laser fluence (*cf.* Section 2.1).

Fig. 2 displays the IR-MPD spectra of cationic and anionic Cu_*x*_O_*y*_(CO_2_) complexes in the 1050–1800 cm^−1^ spectral region which is most diagnostic for the activation of the attached CO_2_. The left column shows that for all cationic complexes two peaks centered at 1225–1278 cm^−1^ and 1372–1393 cm^−1^ (the exact position slightly depends on the cluster size) are observed (indicated by blue dashed lines). The spectral position of these bands is close to the frequency of the characteristic Fermi dyad of the unperturbed CO_2_, which indicates a linear CO_2_ adsorption geometry and only a small perturbation of the CO_2_ molecule upon interaction with the clusters.^44^ Since metal–oxygen vibrations^51^ are typically found below 1100 cm^−1^ the remaining strong bands shown in the IR-MPD spectra most likely arise from motions of non-activated^52–54^ (1400–1550 cm^−1^) or activated^52–57^ (<1200 cm^−1^) O_2_. Due to the large amount of bare clusters as well as CO_2_ complexes in the mass spectrum (with potentially overlapping fragmentation channels) it is not easy to trace fragmentation pathways for all of the clusters. However, for some complexes such as (Cu_2_O_3_(CO_2_)^+^, Cu_2_O_4_(CO_2_)^+^, Cu_3_O_4_(CO_2_)^+^) we find fragmentation *via* loss of O_2_ units indicating the rather weak binding of O_2_ in agreement with the observation of the bands around 1500 cm^−1^ that are indicative for a non-activated O_2_ unit. In addition, a low intensity band at around 1190–1200 cm^−1^ is present. A similar band was previously also observed for Cu_*x*_(CO_2_)^+^ and assigned to the antisymmetric CO_2_ stretching mode (*ν*_3_ = 2349 cm^−1^ for free CO_2_).^38^ This band can potentially become visible at around 1200 cm^−1^ due to the likely presence of second harmonic radiation of the free electron laser.^45^

In marked contrast, the IR-MPD spectra of the anionic complexes (Fig. 2, right column) do not show the Fermi dyad (indicated by blue dashed lines) but instead two bands are observed, centered around 1150 cm^−1^ and 1680 cm^−1^, respectively. These two bands fall in the range where CO_2_ stretching vibrations were observed earlier for bent CO_2_ geometries and thus indicate the activation of CO_2_*via* electron transfer.^30,48–50^ It should be noted that in case of the anionic complexes, the assignment of fragmentation channels is more difficult, but *e.g.* for Cu_2_O_4_(CO_2_)^−^ we have indications that fragmentation also occurs *via* loss of O_2_. Based on these observations we conclude that in the investigated cluster size (number of Cu atoms) and composition (Cu/O ratio) range, cluster charge is the decisive parameter for CO_2_ activation. Although cluster size and composition to some extend affect the reactivity towards CO_2_ (*cf.*Fig. 1) they seem to play only a minor role in the activation of CO_2_.

To gain more insight into the CO_2_ activation and bonding to anionic copper oxide clusters we will in the following discuss the IR-MPD spectra of Cu_2_O_4_(CO_2_)^−^ and Cu_3_O_4_(CO_2_)^−^ in more detail, serving as representatives for all Cu_*x*_O_*y*_(CO_2_)^−^ species.

### 3.3 CO_2_ activation by Cu_2_O_4_^−^ and Cu_3_O_4_^−^ – computations

**Fig. 3:**
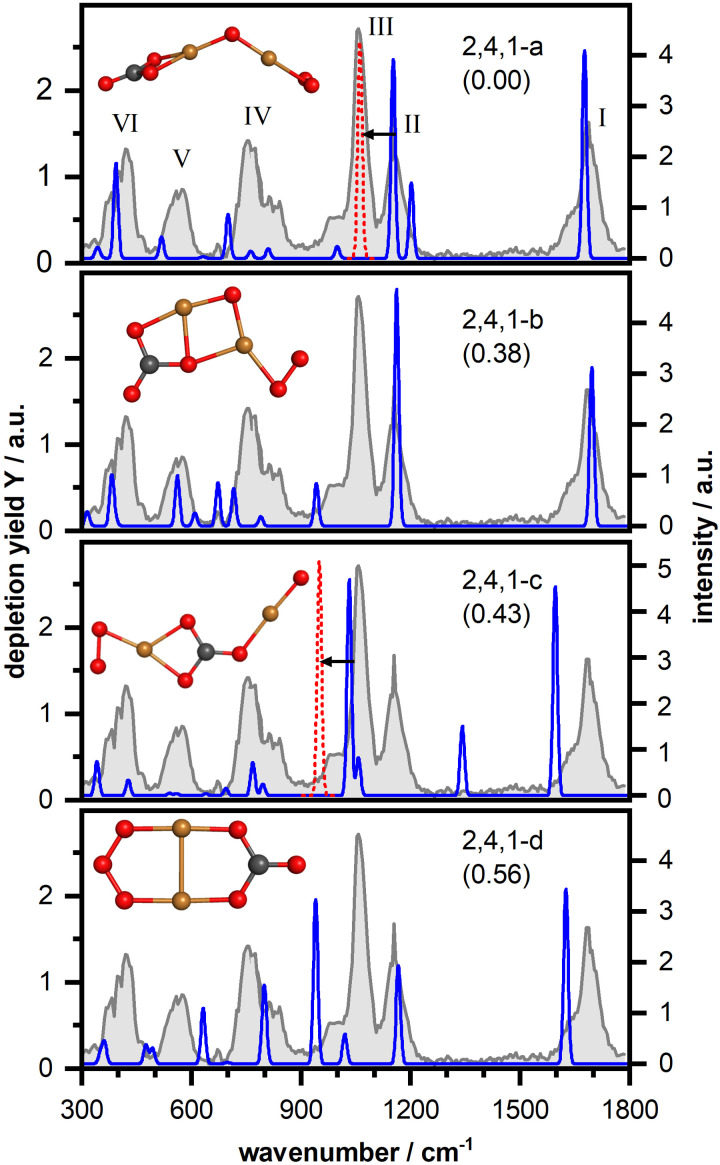
IR-MPD spectrum (in gray) of Cu_2_O_4_(CO_2_)^−^ together with calculated vibrational spectra (in blue) of several isomeric structures (computed relative energies in eV are given between parentheses). The experimental spectrum was measured in two independent runs (1800–660 cm^−1^ and 800–240 cm^−1^, with the former range recorded at reduced FELICE fluence). To obtain an intensity match of the peaks in the overlapping spectral region the depletion yield of the 800–240 cm^−1^ spectrum was multiplied by a factor 0.6. The intensities of the calculated spectrum were scaled the wavelength (*cf.* methods section). The red dashed peak corresponds to the O–O stretching mode resulting from the scaling with a factor of 0.921, the shift is indicated by a black arrow. Cu, C, and O atoms are depicted as yellow, black, and red spheres, respectively.

Fig. 3 displays the IR-MPD spectrum of Cu_2_O_4_(CO_2_)^−^ (gray shaded spectrum) exhibiting six bands (labeled I–VI). As discussed above, bands I (centered at 1681 cm^−1^) and II (1154 cm^−1^) are likely due to vibrations of a bent CO_2_ and band III is likely due to the O–O stretch of a superoxide-like O_2_ unit (1057 cm^−1^, *cf.* 1090 cm^−1^ for free O_2_^−^).^58^ An intense structural search, from which we present the detailed product structures below, revealed that CO_2_ is activated by Cu_2_O_4_^−^*via* exclusive formation of a CO_3_ unit (a formal adduct of an oxide ion and CO_2_), which can bind in different ways to the cluster. It should be noted, that in the structural search also isomers with activated bent CO_2_ bound to one of the Cu atoms were considered. However, such a structure was found to be considerably higher in energy (2.36 eV; *cf.* Fig. S2, ESI†). In the structural search CO_2_ dissociation into carbon monoxide was also considered, but this appears to be unfavorable: the lowest energy isomer containing a copper bound CO was found 3.55 eV higher in energy than the located lowest energy isomer with an intact CO_2_. Furthermore, for all investigated structures the doublet spin state is lower in energy than the quartet. Accordingly, unless otherwise stated, only the doublet state isomers are discussed below.

The lowest energy isomer for Cu_2_O_4_(CO_2_)^−^ (isomer 2,4,1-a in Fig. 3) is based on the bent chain-like O_2_–Cu–O–Cu–O cluster that we identified as the lowest energy isomer of the bare cluster (Fig. S5 (ESI†); a similar but more linear structure has previously been reported^59^) with the CO_2_ molecule forming a CO_3_ unit with the terminal oxygen atom. The resulting CO_3_ group is η^2^(O,O) bound to one of the Cu atoms leading to a stabilization of 1.54 eV relative to the reactants (Cu_2_O_4_^−^ + CO_2_). However, the properties of this metal-bound CO_3_ deviate from those of the unperturbed, highly symmetric (*D*_3h_) carbonate ion. Here, the trigonal planar geometry is distorted with an internal O–C–O angle of 109° as well as a charge of −0.86 e (*cf.* Fig. S5, ESI†), both significantly affecting the vibrational frequencies. Morgan and Staats, in analyzing the IR spectra of copper carbonate dilute solid solution in potassium-halides,^60^ have noted the significant difference between the observed 1785 and 1265 cm^−1^ frequencies and the 1415 cm^−1^ frequency of the doubly degenerate asymmetric stretching vibration of the naked carbonate dianion, attributing the splitting to the lowered symmetry. They tentatively attributed their 1785 cm^−1^ vibration to a C=O stretch in an O=COO resonance structure (the two oxygens bound to C by a single bond interacting with Cu). In agreement, our calculations show that the degenerate E' pair of CO_3_^2−^ (1292 cm^−1^) is split in two components for 2,4,1-a (1202 cm^−1^ and 1676 cm^−1^; see also Fig. S3, ESI†). Considering the different charges in the systems (carbonate dianion and 2,4,1-a monoanion) the numerical frequency values are not directly comparable, but the qualitative similarity is clear. Likewise, the experimental observations of the neutral copper-carbonate in the potassium-halide matrix are not directly comparable with the frequencies of the gas phase anion Cu_2_O_4_(CO_2_)^−^. Nevertheless, qualitatively, it is clear that the observed spectrum is in accordance with the activation of CO_2_, resulting in a distorted CO_3_ structure and the observed frequency near 1700 cm^−1^ is a clear indication for a C=O double bond.

The calculated vibrational spectrum itself is shown in light and dark blue lines in Fig. 3, where the light blue part of the spectrum is vertically scaled by a factor 0.2 to improve visibility. This calculated spectrum provides a very reasonable match for all experimentally observed bands, except for band III, although the frequencies of bands IV–VI seem somewhat underestimated by the calculations. Analysis of the distortion vectors reveals that the modes calculated at 1676 cm^−1^, 1202 cm^−1^, and 998 cm^−1^ correspond to the C–O stretching vibration (matching band I), the asymmetric (band II) and the symmetric (low-frequency shoulder of band III) OCO stretching vibrations of the CO_3_ unit, in accordance with the qualitative discussion above, while the mode at 1152 cm^−1^ arises from the O–O stretching vibration of the η^2^-bound O_2_ unit. All other modes mainly arise from vibrations of the cluster core. Two linear but non planar structures with the same cluster and CO_3_ binding motif were found to be only 0.04 eV and 0.24 eV, respectively, higher in energy (isomers 2,4,1-e and 2,4,1-f – multiplicity 4 – in Fig. S2, ESI†). These isomers have similar vibrational spectra but fail to explain bands V and III.

A second more compact structure (isomer 2,4,1-b, +0.39 eV) consists of a rhombic Cu_2_O_2_ cluster with O_2_ η^2^-bound to one of the Cu atoms and the CO_2_ bridge bound to the Cu–O, forming a CO_3_ unit. This isomer has a similarly good match for bands I (C–O stretching vibration predicted at 1696 cm^−1^) and II (two modes predicted at 1162 cm^−1^ and 1168 cm^−1^ both arising from coupled asymmetric OCO and O–O stretching motions), but fails to reproduce the low-frequency part of the spectrum (mainly vibrations of the cluster core), including band III. We found two more structures with a similar CO_3_ binding motif (isomers 2,4,1-g,h in Fig. S2, ESI†). Both structures are even higher in energy (0.51 eV and 0.83 eV) and the computed harmonic vibrational frequencies and intensities are not in agreement with the IR-MPD spectrum either. Insertion of the CO_2_ molecule into a chain-like Cu_2_O_4_-core results in isomer 2,4,1-c. Such a structure can, however, be excluded based on a mismatch with bands I and II and a rather intense band predicted at 1343 cm^−1^ (asymmetric OCO stretching vibration) which is not observed in the IR-MPD spectrum. Finally, we found an isomer (2,4,1-d; +0.56 eV) containing an ozone-like O_3_ and a CO_3_ unit, for which the vibrational spectrum is largely in disagreement with the experimental spectrum.

Thus, we conclude that the IR-MPD spectrum is best described by isomer 2,4,1-a, although some of the calculated frequencies are shifted compared to the experimental ones. The largest question mark is for the mismatch for band III. To address this, we take a closer look at the O–O stretch vibration, calculated at 1152 cm^−1^. In previous studies it was shown that DFT calculations tend to overestimate the O–O bond strength of η^1^ and η^2^-bound O_2_ units.^52,53,55,61^ For manganese oxide clusters with an experimental O–O stretching vibration around 1200 cm^−1^ a moderate frequency scaling factor of 0.9806 was required to match the experimental data.^55^ For gold oxides experimental O–O stretching vibrations were observed around 1060 cm^−1^ (close to frequency of band III) and significantly stronger scaling (0.907–0.936) was required.^52,53,61^ If we scale the frequency of the O–O stretching vibration of isomer 2,4,1-a by a factor of 0.921 (the average of 0.907 and 0.936) it matches band III very well, as indicated by the red dashed band and black arrow in Fig. 2. In case of isomer 2,4,1-b such a scaling is difficult since both modes predicted at 1162 cm^−1^ and 1168 cm^−1^ are not pure O–O stretching vibrations but arise from coupled asymmetric OCO and O–O stretching motions. For isomer 2,4,1-c scaling clearly leads to an even poorer match. To conclude, we assign the spectrum recorded for the Cu_2_O_4_(CO_2_)^−^ cluster to structure 2,4,1-a, under the assumption that the frequency of the O–O stretching vibration is significantly overestimated by the calculations.

**Fig. 4:**
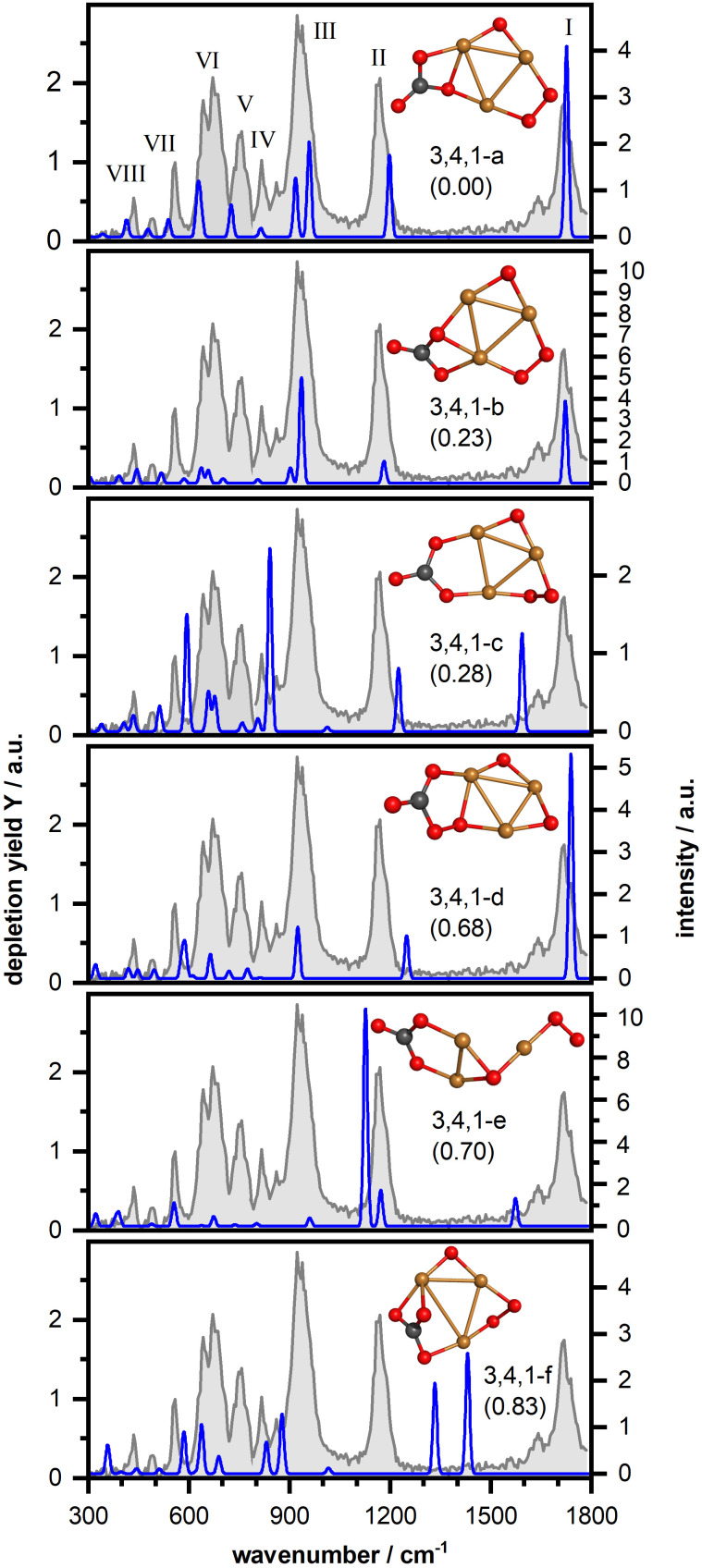
IR-MPD spectrum (in gray) of Cu_3_O_4_(CO_2_)^−^ together with calculated harmonic vibrational spectra (in blue) of several isomeric structures. For more details, see caption of Fig. 3.

Next, we have investigated the complex Cu_3_O_4_(CO_2_)^−^ in more detail. Fig. 4 shows the IR-MPD spectrum (in gray) and the calculated vibrational spectra of selected isomers (more structures and spectra can be found in Fig. S4, ESI†). The IR-MPD spectrum exhibits eight bands (labeled I–VIII). Similar to Cu_2_O_4_(CO_2_)^−^ bands I and II are characteristic CO_2_ bands, but band I is somewhat blue shifted (to 1717 cm^−1^) with respect to the corresponding Cu_2_O_4_(CO_2_)^−^ band. Based on our analysis for Cu_2_O_4_(CO_2_)^−^, band III can be tentatively assigned to the O–O stretching vibration of a super- or even peroxo-like O_2_ unit.

Our structural search revealed thirteen isomeric structures, all differing by an energy of up to about 1 eV and, similar to Cu_2_O_4_(CO_2_)^−^, all containing a CO_3_ unit. Once more, as with Cu_2_O_4_(CO_2_)^−^, an isomer with metal-bound activated CO_2_ is, at 2.34 eV, considerably higher in energy than the lowest energy isomer (*cf.* Fig. S4, ESI†). Again, like for Cu_2_O_4_(CO_2_)^−^, CO_2_ dissociation into CO is energetically unfavorable and the lowest energy isomer containing CO is 3.19 eV higher in energy than the lowest energy isomer 3,4,1-a. Therefore, we will in the following only discuss selected structures with different structural motifs and reference to the ESI† for further similar structures or structures that are considerably higher in energy. Since band III suggests the presence of a (su)peroxo O_2_ we will not discuss any structures here that do not contain such a unit. All structures are found with the triplet spin state lower in energy than the singlet one.

The first class of isomers is based on a planar Cu_3_O_4_^−^ consisting of a triangular Cu_3_ frame with two bridging oxygen atoms and a bridging O_2_ molecule bound to the third Cu–Cu side (*cf.* Fig. S5 (ESI†) for the structure of the bare cluster, which is largely in agreement with the structure previously determined *via* photoelectron spectroscopy^62^) to which a CO_2_ molecule is η^2^(C,O) coordinated forming a μ_2_–η^2^(O,O)-bound CO_3_ unit with one of the bridging oxygen atoms. In the lowest energy isomer (3,4,1-a in Fig. 4) one of the CO_3_ oxygen atoms bridges two Cu atoms and a second O atom is bound to one of the Cu atoms. The calculated vibrational spectrum of this isomer shows a reasonable agreement with the experimental spectrum. However, besides some spectral shifts in the low frequency region as well as for band II, the right part of the double-band VI is missing in the calculated spectrum and the intensity of band III is underestimated. These three bands are better reproduced by isomer 3,4,1-b (with a different position of the CO_3_ group), but now the spectrum in the region of bands V and VII is less favorable. With isomer 3,4,1-c we found a similar structure where the CO_2_ has incorporated the oxygen atom, and the resulting CO_3_ group symmetrically bridges two Cu atoms. This leads to considerable shifts of three high-frequency modes (the terminal C–O stretch, the OCO asymmetric stretch of CO_3_, and the O–O stretch respectively), which disagrees with the experimental spectrum. In particular isomers 3,4,1-a and 3,4,1-b describe band I very well and thus also the blue shift observed with respect to the corresponding Cu_2_O_4_(CO_2_)^−^ band. Since this frequency corresponds to the carbon–oxygen stretching (see Fig. S3, ESI†) the blue shift indicates a strengthening of this bond in Cu_3_O_4_(CO_2_)^−^. Indeed, in the calculated structure of 2,4,1-a, the carbon–oxygen distance is 1.221 Å, against 1.215 Å in 3,4,1-a, with both values well in the C=O double bond range (*ca.* 1.20 Å). Thus, the size of the copper-oxide cluster seems to affect the extent of the CO_2_ activation, which might potentially also affect any subsequent reactions of this unit, for example in a hydrogen reduction reaction. Finally, it should be noted that for all structures in this class, the O–O stretch vibration of the bridging μ_2_-bound O_2_ appears to be considerably better described than in the case of the μ_1_-bound O_2_ of Cu_2_O_4_(CO_2_)^−^.

Other classes of isomers were also found, but their spectral properties do not provide a much better comparison. In the first of these, the CO_2_ binds to the O_2_ unit of the reactant cluster, now forming CO_3_ that coordinates to Cu and O atoms forming a CuOOC(O)O ring (isomer 3,4,1-d; for more see Fig. S4, ESI†). Such a binding motif has a much weaker O–O stretch intensity (very weak mode at 812 cm^−1^) and a blue-shift of the C–O (1739 cm^−1^) and asymmetric OCO stretching vibrations (1248 cm^−1^) and thus an inferior match with the IR-MPD spectrum. In a third class of isomers (isomer 3,4,1-e; for more see Fig. S4, ESI†) the triangular Cu_3_ core is strongly distorted with the CO_3_ unit bridging two Cu atoms and the O_2_ unit η^2^-bound. This leads to a weakening and red-shift of the C–O stretching vibration (1574 cm^−1^) and a considerable blue-shift of the O–O stretching vibration (1126 cm^−1^). An attempt to rationalize the mismatch of the O–O stretch on the same grounds as in the case of Cu_2_O_4_(CO_2_)^−^ (*i.e.* applying a scaling factor of 0.921) results in an even larger mismatch. Finally, a fourth class of isomers found, contains a η^3^-bound CO_3_ group (isomer 3,4,1-f, for more see Fig. S4, ESI†). The additional coordination of the terminal C–O results in a red-shift of its stretching mode by about 300 cm^−1^ and a blue-shift of the OCO asymmetric stretching mode of more than 100 cm^−1^. Both end up in a spectral region where we do not observe any bands in the IR-MPD spectrum, disqualifying this class.

Overall, we conclude that the IR-MPD spectrum is best described by the structures containing a formal CO_3_ group species with a terminal C–O and one oxygen atom bridging two Cu atoms, such as isomers 3,4,1-a or 3,4,1-b. Since none of these isomers provide a perfect match, we hesitate to make a firm assignment but assume that multiple, quite similar species are present in the molecular beam. This assumption is supported by the small energy difference between isomers 3,4,1-a, -b, and -c raising the possibility of a frustrated rotation of the formal CO_3_ unit linking the different structures. We finally would like to note that there is no ready explanation for the low frequency satellite of band I (which also appears to be present for other anionic clusters shown in Fig. 2). One possibility would be an overtone of the O–O stretch (found at 930 cm^−1^), but the required anharmonicity exceeding 20% seems too much. Another possibility would be a second isomer with a slightly differently bound CO_2_. However, as discussed above for Cu_3_O_4_(CO_2_)^−^ no such isomer was located. Therefore, it can be speculated that the weak satellite bands could be hot bands, originating from vibrationally excited states insufficiently cooled during the expansion of the molecular beam into the vacuum. It is known that vibrational cooling is poor in any molecular beam expansion and especially for those from a flow-tube, where pressures are already lower than stagnation pressures of conventional molecular beams.

## 4 Summary and conclusions

We have produced cationic and anionic copper oxide clusters *via* laser ablation of a ^65^Cu target in the presence of an O_2_/He gas pulse. Under the given experimental conditions, the cluster distribution of cationic clusters is dominated by highly oxidized clusters, while mainly stoichiometric and oxygen-deficient anionic clusters were formed. Subsequent reaction of the formed clusters with CO_2_ in a flow tube reactor showed that primarily near-stoichiometric Cu_*x*_O_*y*_(CO_2_)^+/−^ complexes are formed. Furthermore, IR-MPD spectroscopy of the formed products in conjunction with DFT calculations on two selected systems (Cu_2_O_4_(CO_2_)^−^ and Cu_3_O_4_(CO_2_)^−^) revealed that (1) CO_2_ binds to cationic clusters as a linear molecule without noteworthy activation, while activation occurs on all anionic clusters. Thus, in the investigated size range, cluster charge is the decisive parameter for CO_2_ activation and size or composition (Cu/O ratio) only play a minor role. (2) activation of CO_2_ upon binding to anionic copper oxide clusters leads to formation of a CO_3_ unit. The activation of CO_2_ is fine-tuned by the size of the copper oxide cluster influencing the strength of the C=O bond in the CO_3_ group. (3) CO_2_ dissociation is highly unlikely due to the relatively weak binding of CO to the copper oxide clusters.

These findings are important for the potential rational design of copper-based CO_2_ hydrogenation catalysts. Our study shows that anionic copper oxide clusters are able to activate CO_2_, but that the most dominant factor is the presence of excess negative charge, something which was already found by Weber and co-workers for single-atomic ions,^47,50^ and later confirmed by others for few-atom metal clusters.^48,49,63,64^ Furthermore, we observe the exclusive formation of a CO_3_ unit while dissociation to CO is energetically unfavorable. This indicates that the presence of oxygen atoms stabilizes CO_3_ and prevents CO_2_ dissociation. Similar CO_3_ intermediates have previously been observed for some anionic mono^65–67^- and few-metal oxide clusters^64^ as well as (basic) metal-oxide surfaces^68–71^ and even a few cationic species forming a CO_3_ intermediate have been reported.^72,73^ The presence of CO_3_ and the lack of CO_2_ dissociation suggest that any further hydrogenation reaction proceeds *via* hydrogenation of the activated CO_2_ (CO_3_), potentially leading to formate or bicarbonate intermediates. Consequently, the formation of carbon monoxide is less likely. As carbon monoxide is a key intermediate in the C_2+_ product formation route,^74,75^ we speculate that C_1_ products are more likely to form (*via* the formate route) which can lead to methane or methanol.^76–78^

## Data availability

The data for this article are shown in the Figure. Further data supporting this article have been included as part of the ESI.†

## Conflicts of interest

There are no conflicts to declare.

## Supplementary Material

CP-026-D4CP02651A-s001
