# Supplementary material for: CO2 activation by copper oxide clusters: size, composition, and charge state dependence
Source: Phys Chem Chem Phys. 2024 Sep 2;26(36):24126–34. doi: 10.1039/d4cp02651a (PMC11385096; doi:10.1039/d4cp02651a)
Supplement: CP-026-D4CP02651A-s001 [file CP-026-D4CP02651A-s001.pdf]

Supplementary Information (SI) for Physical Chemistry Chemical Physics.
This journal is © the Owner Societies 2024

Supporting information to

CO_2 Activation by Copper Oxide Clusters: Size, Composition, and Charge State Dependence

Pavol Mikolaj, Barbara Zamora Yusti, László Nyulászi, Joost M. Bakker, Tibor Hölzl, Sandra M. Lang.

S1 Intensity scaling of calculated spectra

We find that multiplying the intensities of the calculated spectra by the wavelength λ gives a better match between the calculated and experimental spectra, as shown in Figure S1. We speculate that the better match is caused by a wavelength dependent flux. Given that the intracavity laser's spatial mode is quite well-defined, we conclude that the frequency dependence of the photon flux is not due to a changing of the beam size but due to an increase in spectral brightness. The assumed Gaussian laser mode's cross-section is proportional to the wavelength λ and consequentially, because the macropulse energy is relatively constant over a scanned wavelength range, the fluence (energy per unit area) scales with 1/λ, but the photon flux (photons per unit area) does not, because division by the photon energy (hν=hc/ λ) cancels the 1/λ dependence. Instead, because the laser's spectral bandwidth is proportional to the wavenumber, the flux per unit wavenumber reduces with wavenumber. Therefore, the intensity of all spectra shown in the manuscript and the supporting information were scaled by we scaled by λ.

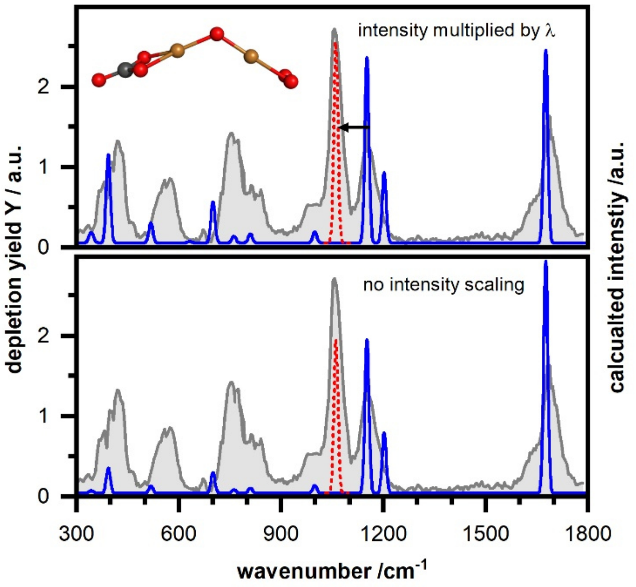

Figure S1: IR-MPD spectrum (gray) of $Cu_2O_4(CO_2)^-$ together with the calculated infrared spectrum (blue) of isomer 2,4,1-a. In the upper panel the intensity of the calculated spectrum was scaled with the l, while in the lower spectrum the intensity was not scaled.

S2 Additional structures and vibrational spectra for $Cu_2O_4(CO_2)^-$

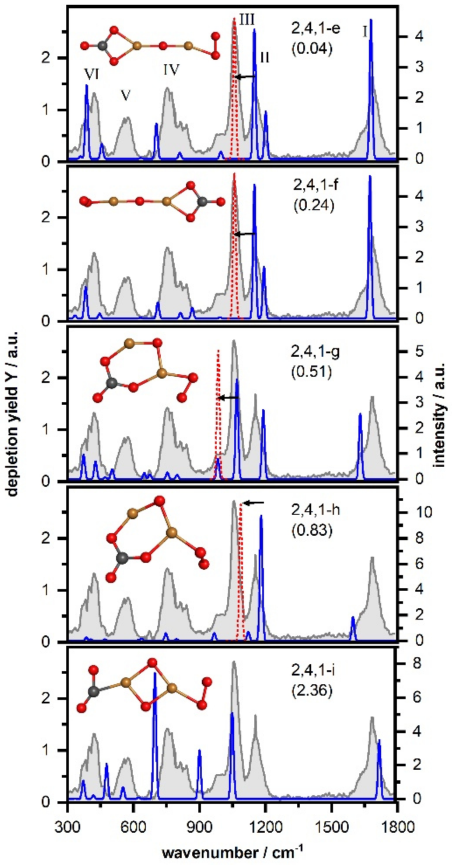

Figure S2: IR-MPD spectrum (in gray) of $Cu_2O_4(CO_2)^-$ together with calculated vibrational spectra (in blue) of several isomeric structures (relative energies are given in brackets in eV). The experimental spectrum was measured in two independent runs (1800-660 cm^{-1} and 800-240 cm^{-1}, while the first one was obtained at reduced FELICE fluence). To obtain an intensity match of the peaks in the overlapping spectral region the intensity of the 800-240 cm^{-1} spectrum was multiplied by a factor 0.6. The intensities of the calculated spectrum were mutliplied by the wavelangth. The red dashed peak corresponds to the O-O stretching mode resulting from the scaling with a factor of 0.921. Isomers **2,4,1-e** and **2,4,1-f** are similar to isomer **2,4,1-a** shown in the main text and also exhibit similar vibrational spectra. Isomers **2,4,1-g** and **2,4,1-h** provide an inferior match with the IR-MPD spectra, in particular for band I. Cu, C, and O atoms are depicted as yellow, black, and red spheres, respectively.

Table 1: Calculated frequencies and (unscaled) intensities of the $Cu_2O_4(CO_2)^-$ isomers shown in Figure 3 of the main text.

isomer	frequency / cm^{-1}	intensity / km mol^{-1}	isomer	frequency / cm^{-1}	intensity / km mol^{-1}	isomer	frequency / cm^{-1}	intensity / km mol^{-1}
**2,4,1-a**	1676.27	687.87	**2,4,1-b**	1696.25	530.51	**2,4,1-c**	1596.18	724.75
	1202.48	179.67		1168.34	32.44		1342.10	202.19
	1151.92	452.85		1161.71	526.74		1056.70	86.96
	998.62	23.30		941.69	78.54		1031.59	484.47
	809.75	15.42		789.49	14.58		795.12	20.17
	761.85	11.08		715.58	52.66		767.47	55.35
	700.16	60.74		672.41	57.36		693.82	10.79
	631.76	2.072		608.97	15.57		639.34	3.09
	518.00	21.84		561.51	55.93		559.19	2.70
	393.28	73.87		392.16	4.55		539.89	2.98
	348.49	2.49		381.88	37.91		426.94	14.80
	341.14	6.15		314.89	8.88		341.33	25.30
	243.19	1.65		246.05	20.16		274.45	10.78
	206.67	3.05		245.42	7.84		236.19	6.97
	178.39	12.48		170.03	3.84		198.50	0.10
	144.59	6.03		156.14	6.13		182.22	6.81
	93.06	0.09		117.63	3.69		158.38	1.41
	80.87	3.52		107.07	0.02		151.99	23.37
	59.12	2.64		91.54	1.51		99.71	0.01
	45.94	0.84		75.14	1.73		72.57	3.92
	28.91	1.75		58.51	0.02		63.44	2.22
**2,4,1-d**	1625.44	560.76						
	1166.77	227.13						
	1019.43	60.17						
	939.75	305.44						
	805.43	11.27						
	798.76	118.26						
	766.29	0.23						
	697.86	1.66						
	631.85	68.89						
	493.27	15.19						
	475.38	18.11						
	362.09	14.75						
	351.09	7.25						
	243.48	8.34						
	227.19	0.24						
	195.51	11.23						
	150.83	0.48						
	134.57	1.87						
	109.88	19.77						
	94.06	18.0						
	76.39	4.67						

S3 Calculated vibrational spectra for $Cu_3O_4(CO_2)^-$ and isolated CO_3^{2-}

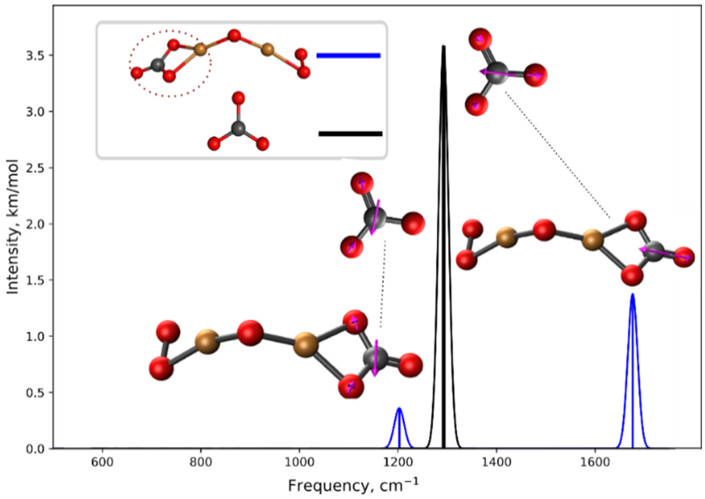

Figure S3: DFT calculated harmonic characteristic frequencies of the CO_3 unit in $Cu_2O_4(CO_2)^-$ (blue) together with calculated vibrational spectra of an isolated carbonate ion CO_3^{-2} (black). Cu, C, and O atoms are depicted as yellow, black, and red spheres, respectively.

S4 Additional structures and vibrational spectra for $Cu_3O_4(CO_2)^-$

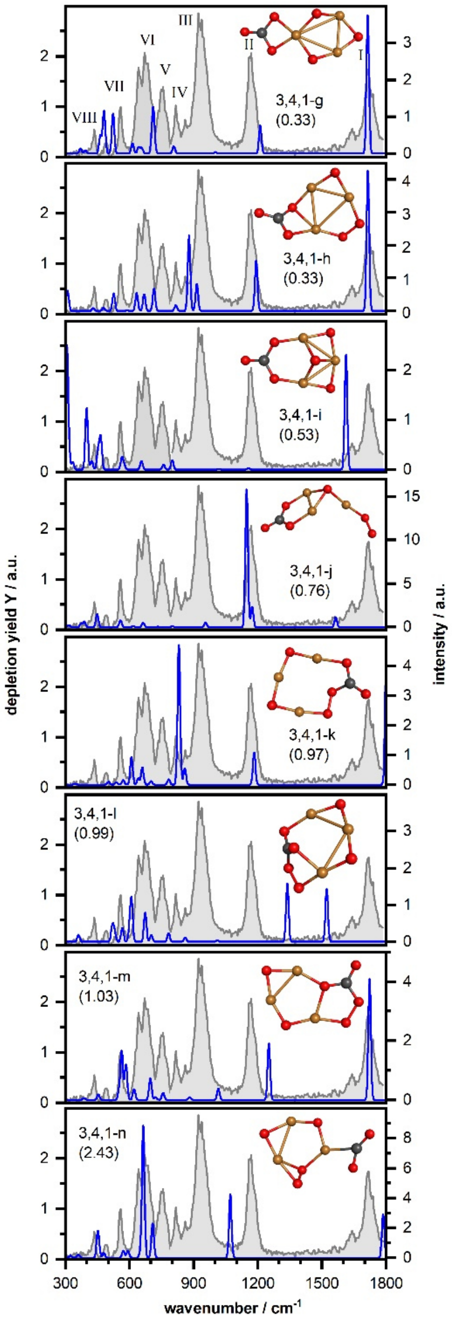

Figure S4: IR-MPD spectrum (in gray) of $Cu_3O_4(CO_2)^-$ together with calculated vibrational spectra (in blue) of several isomeric structures (relative energies are given in brackets in eV). The experimental spectrum was measured in two independent runs (1800-660 cm^{-1} and 800-240 cm^{-1}, while the first one was obtained at reduced FELICE fluence). To obtain an intensity match of the peaks in the overlapping spectral region the intensity of the 800-240 cm^{-1} spectrum was multiplied by a factor 0.6). The intensities of the calculated spectrum were multiplied by the wavelength. Isomer **3,4,1-h** is similar to isomers **3,4,1-a,b** of the main text; isomer **3,4,1-j** is similar to isomer **3,4,1-e**; isomers **3,4,1-k-m** correspond to the same isomer class as **3,4,1-e**. Isomers **3,4,1-g,i** do not contain an O_2 unit. While the co-existence of some of these isomers in the molecular beam cannot be completely excluded, none of them provide a superior fit as those discussed in the main text. Cu, C, and O atoms are depicted as yellow, black, and red spheres, respectively.

Table 2: Calculated frequencies and (unscaled) intensities of the $Cu_3O_4(CO_2)^-$ isomers shown in Figure 4 of the main text.

isomer	frequency / cm^{-1}	intensity / km mol^{-1}	isomer	frequency / cm^{-1}	intensity / km mol^{-1}	isomer	frequency / cm^{-1}	intensity / km mol^{-1}
**3,4,1-a**	1725.88	710.69	**3,4,1-b**	1722.10	672.85	**3,4,1-c**	1593.13	201.58
	1197.75	212.25		1181.46	124.12		1224.81	99.46
	958.52	195.66		935.03	470.77		1012.25	5.54
	917.83	116.73		902.25	66.43		841.20	198.01
	813.99	14.93		805.23	15.82		805.28	13.41
	725.56	50.24		701.57	16.50		758.57	8.38
	635.49	35.97		657.36	41.28		676.79	30.50
	626.25	60.69		636.32	47.71		657.98	33.89
	620.99	1.60		585.07	13.38		593.46	89.89
	538.71	20.61		517.48	26.34		512.31	16.72
	478.16	7.95		444.55	29.80		433.96	8.93
	413.92	15.32		391.18	14.39		405.68	4.61
	342.97	2.21		303.53	9.20		339.06	3.17
	282.78	5.12		287.98	2.25		277.16	8.11
	234.06	0.68		255.12	0.49		242.08	11.83
	211.48	5.54		239.16	4.64		219.76	13.54
	199.46	10.65		203.40	8.12		199.12	3.96
	184.47	3.63		180.80	7.29		184.70	9.19
	160.83	5.65		148.66	4.97		153.34	8.30
	124.31	2.77		138.60	0.68		127.24	3.23
	118.21	3.70		129.99	1.91		108.63	1.40
	96.16	3.48		109.82	2.19		94.32	2.05
	78.17	2.15		87.11	0.67		75.95	0.14
	59.30	2.87		43.61	6.37		60.93	4.02
**3,4,1-d**	1738.88	928.05	**3,4,1-e**	1573.53	208.95	**3,4,1-f**	1430.85	372.17
	1249.05	128.14		1171.73	200.42		1333.36	259.36
	924.27	114.39		1126.37	1160.57		1015.8	12.54
	811.86	2.07		960.05	39.25		877.13	112.31
	774.08	18.59		801.45	10.88		830.79	57.24
	718.98	13.35		737.08	6.16		688.67	26.20
	663.73	38.71		673.57	31.79		637.41	67.74
	611.02	4.19		637.37	2.54		622.27	3.08
	588.40	44.94		555.36	61.84		584.91	53.24
	578.23	26.88		488.72	5.47		511.41	5.28
	495.94	10.66		390.70	23.91		443.32	5.03
	447.18	9.33		379.85	13.06		397.64	1.42
	419.40	10.57		321.81	19.49		357.61	22.24
	321.05	10.88		281.06	2.85		286.46	2.56
	263.14	3.80		200.53	45.44		216.90	6.65
	235.79	21.15		176.55	6.65		190.17	14.44
	216.95	4.83		165.53	7.61		167.56	13.12
	186.85	5.87		147.00	2.98		152.24	4.82
	166.93	1.25		145.47	9.91		113.92	6.97
	158.11	2.20		93.87	0.46		92.35	2.46
	130.60	7.53		78.02	18.46		86.55	4.32
	108.05	4.56		54.61	1.98		79.37	4.20
	78.21	0.31		48.58	3.71		73.65	1.42
	62.22	1.91		31.67	4.06		46.23	1.36

S5 Structures and relative energies of anionic $Cu_2O_4^-$ and $Cu_3O_4^-$ clusters

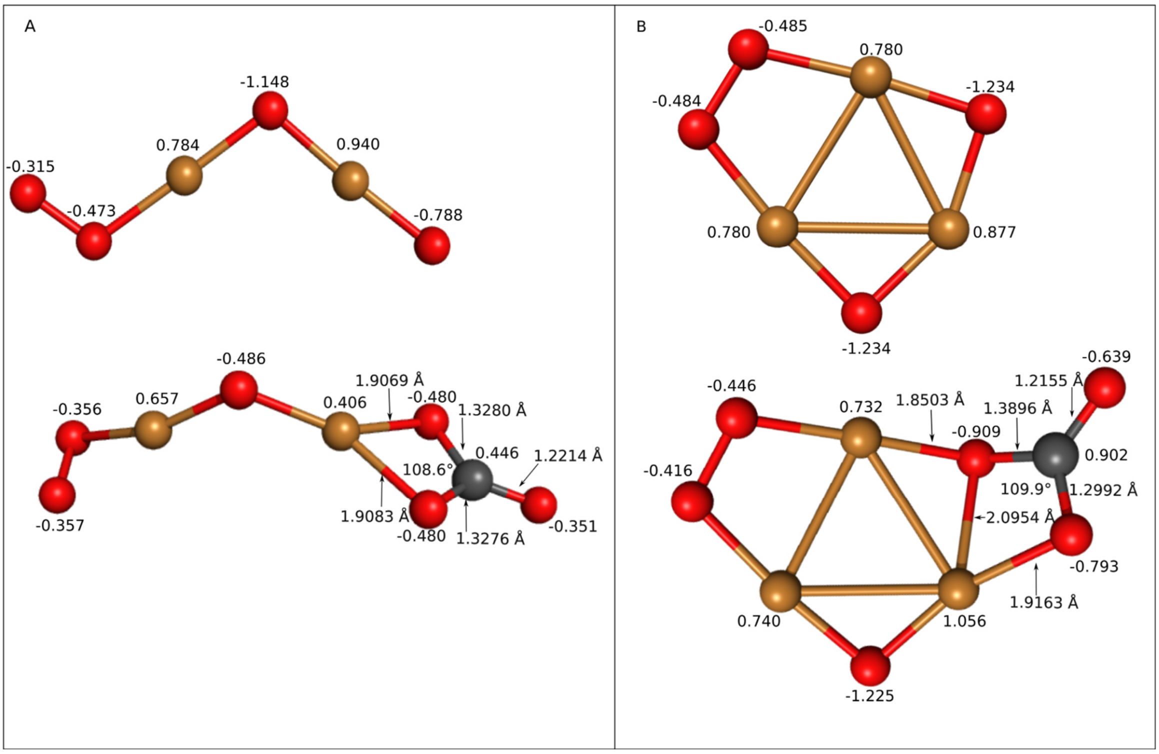

Figure S5: Natural atomic charges (e), C-O and O-Cu bond lengths (Å) on the carbonates, and O-C-O angle for the (A) **2,4,1-a** isomer of $Cu_2O_4(CO_2)^-$ and the **3,4,1-a** isomer of $Cu_3O_4(CO_2)^-$ (B) as well as their respective bare clusters (top). Cu, C, and O atoms are depicted as yellow, black, and red spheres, respectively.

S6 XYZ coordinates

$Cu_2O_4^-$ / $Cu_3O_4^-$

$Cu_2O_4^-$	$Cu_3O_4^-$
**2,4-I**	**3,4-I**
Cu 1.777073464 0.062893086 0.001998914	Cu 0.6963040039 -1.2845468863 0.1349027997
Cu -1.037518197 0.151676540 -0.008304471	Cu -1.6354260762 -0.3286274201 0.0108704248
O 3.154177805 -0.949323325 0.006198118	Cu 0.3068376905 1.2815319510 -0.0039280752
O 0.419718595 1.158914112 0.002574133	O 2.3396546852 -0.4294147647 0.0651954742
O -3.681699504 -0.119558065 0.025705414	O -1.4642293921 1.4510570802 -0.0014679473
O -2.573084741 -0.867847615 -0.011620022	O 2.1302210385 0.9449038804 -0.0164702291
	O -0.9450975803 -1.9692832536 0.1691095740
SCF energy (Eh) = -3582.036028	SCF energy (Eh) = -5222.665392
Multiplicity = 2	Multiplicity = 3

$Cu_2O_4(CO_2)^-$

**2,4,1-a**<br><br>Cu 2.070549369 0.149988126 0.001840883<br>Cu -1.019037234 0.405136079 0.028897802<br>O 0.626741811 1.112102747 0.064315256<br>O 3.659472803 -0.822528519 0.621077173<br>O 3.641419373 -0.800282021 -0.709239715<br>O -2.475716876 -0.257788697 1.065597002<br>C -3.211992239 -0.431709873 -0.025788207<br>O -2.505050598 -0.044481774 -1.080640598<br>O -4.349603824 -0.875564577 -0.053195695<br>SCF energy (Eh) = -3770,759571 (M=2)	**2,4,1-b**<br><br>Cu 1.235004827 -0.008461059 -0.000718381<br>Cu -1.033175076 1.069126945 0.002014875<br>O 0.615565780 1.720481505 0.004089456<br>O 3.236534867 -0.251845038 -0.009049471<br>O 2.610421048 -1.416823645 0.004280466<br>O -2.623923098 0.053097452 -0.012954265<br>C -1.978260501 -1.091351295 -0.001207358<br>O -2.464137518 -2.208644577 0.001790755<br>O -0.622398551 -0.922666062 0.008048788<br>SCF energy (Eh) = -3770,745533 (M=2)<br>SCF energy (Eh) = -3770.7385576957 (M=4)	**2,4,1-c**<br><br>Cu 2.812908679 0.036066516 -0.000449585<br>Cu -2.044671299 0.050437419 0.002550500<br>O -0.221923032 0.594111115 -0.015868803<br>O 1.304681358 -1.085506280 -0.008957150<br>O 4.267495255 0.992473120 0.013192469<br>O -3.874400447 -0.075299203 0.011674518<br>C 0.121268784 -0.667523194 -0.006747627<br>O -0.908190227 -1.472908008 0.004835744<br>O -3.443474998 1.234194890 -0.007431877<br>SCF energy (Eh) = -3770,743935 (M=2)<br>SCF energy (Eh) = -3770.73178035441 (M=4)
**2,4,1-d**<br><br>Cu -0.217977662 -1.198449658 -0.011845321<br>Cu -0.210751310 1.198681987 0.012337820<br>O 1.606292257 -1.151070122 0.017012547<br>O -2.772034735 0.004250957 0.000620036<br>O -2.059693950 1.179041049 -0.025517997<br>O 1.613927775 1.150574716 -0.022147502<br>C 2.286543745 -0.002371542 -0.000385333<br>O 3.517063234 -0.006625476 0.003092994<br>O -2.066319865 -1.175234656 0.025443612<br><br>SCF energy (Eh) = -3770,739200 (M=2)	**2,4,1-e**<br><br>Cu -1.192598656 0.000598902 -0.002387689<br>Cu 2.306230411 -0.002281762 -0.000440255<br>O 0.579919294 -0.005627382 -0.002556487<br>O -2.759572841 -1.079270657 0.007724957<br>O -2.758757386 1.080191000 -0.006382610<br>O 4.152039347 0.670754379 0.014256989<br>C -3.533718416 0.000866600 0.002318047<br>O -4.754864271 0.001301125 0.004004848<br>O 4.154609556 -0.661898046 -0.008534937<br><br>SCF energy (Eh) = -3770,757954 (M=2)	**2,4,1-f**<br><br>Cu 1.191544768 0.005480761 0.003237425<br>Cu -2.312690812 0.007139963 -0.007726376<br>O -0.588338681 0.021302685 -0.007056229<br>O 2.780737635 1.080000281 -0.032106405<br>O 2.767822525 -1.087876212 0.043778127<br>O -4.156940524 0.044095418 0.671216766<br>C 3.544087942 -0.008463165 0.000496185<br>O 4.766399832 -0.015810714 -0.008241670<br>O -4.163592335 -0.081114209 -0.651690279<br><br>SCF energy (Eh) = -3770,750652 (M=4)
**2,4,1-g**<br><br>Cu 1.147326403 -0.079695991 -0.054783764<br>Cu -1.118280688 1.240131326 0.138224136<br>O -0.342763128 -1.266308786 -0.035154671<br>O 2.590384061 -1.245389187 0.181213128<br>O 2.997395298 0.046871747 0.062334015<br>O -2.310094688 -0.102262022 -0.064644935<br>C -1.659814821 -1.281471946 -0.046150837<br>O -2.296515275 -2.330022738 -0.042667899<br>O 0.501164132 1.651636858 -0.368937856<br>SCF energy (Eh) = -3770,740791 (M=2)<br>SCF energy (Eh) = -3770.73359106221 (M=4)	**2,4,1-h**<br><br>Cu 1.036859449 0.374002410 0.256104262<br>Cu -1.339657002 1.024298263 -0.257362404<br>O 0.026448643 1.929432145 0.366350203<br>O 3.194491387 -0.916318843 -0.884731701<br>O 2.764030520 -0.358726997 0.193413024<br>O -2.185021465 -0.588329582 -0.216033695<br>C -1.314346250 -1.554065291 0.088474665<br>O -0.041931124 -1.242362099 0.298421213<br>O -1.674617141 -2.726985593 0.180785720<br>SCF energy (Eh) = -3770,729289 (M=2	**2,4,1-i**<br><br>Cu 1.585099796 -0.023361157 0.001729449<br>Cu -1.140460376 0.260223833 0.006525892<br>O 0.057282370 -1.146065861 0.034186314<br>O 3.385431287 -0.996990162 -0.020924094<br>O 3.653021152 0.386630009 0.014000515<br>O 0.398049363 1.473876033 -0.047717345<br>O -3.071618815 0.968529987 0.029547364<br>C -3.045774484 -0.312355254 -0.003365813<br>O -3.749652393 -1.310340766 -0.036494008<br>SCF energy (Eh)= -3770.67274999 (M=2)

$Cu_3O_4(CO_2)^-$

**3,4,1-a**<br><br>Cu 0.148143263 -1.299935880 -0.181464177<br>Cu -0.783936637 1.214224254 -0.030091689<br>Cu 1.781500756 0.679290417 0.048295117<br>O 2.718578124 -0.881952063 0.356896753<br>O -2.503709052 0.753634322 0.678841953<br>O 1.835870805 -1.936925471 0.287641820<br>O -1.499161093 -0.637187343 -0.701887374<br>C -2.600971335 -0.412611239 0.114520175<br>O -3.504627169 -1.221215195 0.197582817<br>O 0.750587623 2.081381062 -0.313145883<br>SCF energy (Eh) = -5411.375208 (M=3)<br>SCF energy (Eh) = -5411,353506 (M=1)	**3,4,1-b**<br><br>Cu 0.090543012 -1.515699033 0.059188937<br>Cu -1.933282045 -0.114859706 -0.113175265<br>Cu 0.432781543 0.930654536 0.197190740<br>O 3.751976304 -0.423391696 -0.348644044<br>O -2.101546932 1.709462041 -0.072361901<br>O 1.654141187 -0.582236224 0.508502172<br>O -1.650382433 -1.862832009 -0.174557545<br>C 2.681516307 0.106023766 -0.124764806<br>O 2.308685964 1.327370726 -0.358268674<br>O -0.862915419 2.289262071 0.019787600<br>SCF energy (Eh) = -5411.366843 (M=3)<br>SCF energy (Eh) = -5411,353208(M=1)	**3,4,1-c**<br><br>Cu 1.911920630 0.381037866 0.102697874<br>Cu -0.497046324 1.341797209 -0.044477185<br>Cu -0.092166988 -1.205443399 -0.154362344<br>O 2.435175584 -1.349567424 0.618536733<br>O -1.846004212 -1.251888650 0.276075559<br>O 1.696338489 -1.459664400 -0.640345354<br>O 1.179372019 1.982212742 -0.047364115<br>C -2.734968864 -0.279379410 0.111480416<br>O -2.270722079 0.924165895 -0.144524241<br>O -3.937747179 -0.511268431 0.202524602<br>SCF energy (Eh) =-5411.365077 (M=3)<br>SCF energy (Eh) = -5411,358944 (M=1)

3,4,1-d

Cu 0.447171484 -1.117032267 -0.159246899
Cu -2.172956307 -0.391917314 0.146753694
Cu -0.548623970 1.336288624 -0.065838218
O 2.270002932 1.097105480 -0.395777574
O -2.237704542 1.342763604 0.594579230
O 0.959486851 0.704425077 -0.963292065
O -1.201969853 -1.824591468 -0.224501120
C 2.902403768 0.048412010 0.270705518
O 2.250797953 -1.047810525 0.411842614
O 4.027315709 0.317694795 0.658071188
SCF energy (Eh) = -5411.350063 (M=3)
SCF energy (Eh) = -5411,333856 (M=1)

3,4,1-e

Cu -0.787230604 -1.317891166 0.027735738
Cu -0.483259881 0.783315581 0.717374715
Cu 1.941993187 0.062792327 0.051480301
O 0.660967677 -0.681433829 1.085995186
O -2.089461887 1.365826402 0.003473405
O 3.293598276 0.859205076 -0.924706950
O -2.431435631 -0.819856477 -0.645576044
C -2.845915203 0.447984399 -0.586043161
O -3.933264311 0.753155321 -1.075885009
O 4.199834985 -0.102670480 -0.891409698
SCF energy (Eh) = -5411.349559 (M=3)
SCF energy (Eh) = -5411,336083 (M=1)

3,4,1-f

Cu 1.384870935 0.992903245 0.000250677
Cu -1.262060131 1.222652741 -0.073723878
Cu 0.410871732 -1.648005220 -0.096615713
O 2.055092504 -0.915254514 -0.538544110
O -1.575884607 -0.216512200 1.278860716
O -1.431745183 -2.113836939 0.052051180
O 0.145651737 2.302679560 -0.122890419
C -1.845460628 -0.897190092 0.197709126
O -2.430217787 -0.231780017 -0.749300267
O 2.686599611 -0.209774848 0.548113371
SCF energy (Eh) = -5411.344794 (M=3)
SCF energy (Eh) = -5411,334852 (M=1)

3,4,1-g

Cu 1.573204764 1.122945363 0.117808079
Cu -1.001528477 0.090938481 -0.316757792
Cu 1.764411659 -1.218898206 0.130879362
O -2.394174612 -1.135288643 -0.027596678
O 0.271306138 -1.171886838 -0.810732650
O 2.978218129 0.094979532 0.525671515
O 0.020053086 1.617232446 -0.518436161
C -3.216526856 -0.149752591 0.304533306
O -4.381813756 -0.270188058 0.633027139
O -2.549512644 0.995643069 0.216421876
SCF energy (Eh) = -5411.362964 (M=3)
SCF energy (Eh) = -5411,34261 (M=1)

3,4,1-h

Cu -0.512734409 -1.238592271 -0.107771034
Cu -0.160328964 1.419818694 -0.202313394
Cu 1.977655618 0.282217529 0.176793555
O 1.083043730 -2.138600394 -0.351725905
O 1.456368816 1.972324986 0.337290871
O 2.262561741 -1.518076281 0.070857382
O -1.642860069 0.458078872 -0.715756398
C -2.623478374 0.021983861 0.161496477
O -2.244623474 -1.085855663 0.726982489
O -3.676028850 0.615656257 0.294408620
SCF energy (Eh) =-5411.362938 (M=3)
SCF energy (Eh) = -5411,341266 (M=1)

3,4,1-i

Cu 1.912408770 -0.000646930 -0.228404311
Cu -0.139911755 1.410074306 0.102014693
Cu -0.142559429 -1.410909052 0.101641992
O -1.950794785 -1.141181688 0.003260096
O 0.494049410 -0.000590361 1.209375112
O 1.622319459 -1.785543458 -0.280252733
O 1.624665712 1.785922674 -0.277812149
C -2.604044455 0.001251580 -0.160328567
O -1.946192218 1.141635654 0.000054519
O -3.799537988 0.004189570 -0.444668276

SCF energy (Eh) =-5411.355750 (M=3)
SCF energy (Eh) = -5411,346655 (M=1)

3,4,1-j

Cu -0.837070635 1.278782155 -0.252806745
Cu -0.433876041 -0.560347068 0.942864391
Cu 1.928155663 0.282060750 0.017488159
O 3.806768684 -1.591184725 -0.762542846
O 0.590755856 1.033429077 0.983843470
O 3.374085914 -0.367093233 -0.912854723
O -1.935036518 -1.422053939 0.303671462
C -2.731029565 -0.731576981 -0.506528340
O -2.380865717 0.490813426 -0.897989702
O -3.789818621 -1.222025280 -0.899084951
SCF energy (Eh) = -5411.347217 (M=3)
SCF energy (Eh) = -5411,322584 (M=1)

3,4,1-k

Cu -0.876667915 -1.660334374 0.095042684
Cu -1.768798492 0.723722146 -0.144393937
Cu 0.768935195 1.517543389 0.087937906
O 1.397025782 -0.775186940 0.599283721
O -0.866088096 2.208221126 0.217716358
O 2.546595117 0.919066256 -0.139413738
O -2.423811870 -0.925996555 -0.355404587
C 2.557171231 -0.375163003 -0.132254124
O 3.346827147 -1.162231840 -0.588572332
O 0.883999137 -2.088375253 0.225704553
SCF energy (Eh) =-5411.339581 (M=3)
SCF energy (Eh) = -5411,319968 (M=1)

3,4,1-l

Cu 1.489204509 -1.191613127 0.011544882
Cu 1.163273664 1.352633715 0.081825367
Cu -1.532378496 0.898417275 -0.042824597
O -2.775290473 -0.421544177 0.518774238
O -0.359354059 2.240607859 -0.161056845
O -0.874083644 -0.658442614 -1.143604153
O 2.538671376 0.242273910 0.285379418
C -0.910260729 -1.571388042 -0.266860335
O -1.923186386 -1.627169128 0.607457872
O 0.015577405 -2.437647076 -0.090033268
SCF energy (Eh) =-5411.338838 (M=3)
SCF energy (Eh) = -5411,321741 (M=1)

3,4,1-m

Cu 0.651327361 -1.321320041 -0.001290297
Cu -2.085886796 -0.470007865 0.003315183
Cu -0.683045774 1.429862915 -0.002013984
O 2.512005505 -1.418100770 -0.002450649
O -2.507354305 1.268117251 -0.003847630
O 1.040847551 0.653135825 -0.002868114
O -1.051817658 -1.898545676 0.000653565
C 2.319572479 0.961418529 0.001751862
O 3.154047761 -0.099026367 0.000804453
O 2.788910671 2.083666430 0.006354955
SCF energy (Eh) = -5411.337455 (M=3)
SCF energy (Eh) = -5411,333901(M=1)

3,4,1-n

Cu 1.173133532 -0.149433882 -0.370114381
Cu -1.087293425 1.537423803 0.057610757
Cu -1.796539097 -0.895482425 0.201257328
O -0.503099374 -2.303607301 0.441183566
O -2.701919030 0.667961960 0.212397574
O -0.241655298 -1.539804186 -0.853799711
O 0.617826041 1.605318849 -0.545337004
C 3.073719195 0.039075425 0.355751857
O 2.954221582 -1.196937566 0.131915354
O 3.770620526 0.952421999 0.750094150

SCF energy (Eh) = -5411.28606312 (M=3)

S7 References

[34]
M. J. Frisch, G. W. Trucks, H. B. Schlegel, G. E. Scuseria, M. A. Robb, J. R. Cheeseman, G. Scalmani, V. Barone, G. A. Petersson, H. Nakatsuji, X. Li, M. Caricato, A. V. Marenich, J. Bloino, B. G. Janesko, R. Gomperts, B. Mennucci, H. P. Hratchian, J. V. Ortiz, A. F. Izmaylov, J. L. Sonnenberg, Williams, F. Ding, F. Lipparini, F. Egidi, J. Goings, B. Peng, A. Petrone, T. Henderson, D. Ranasinghe, V. G. Zakrzewski, J. Gao, N. Rega, G. Zheng, W. Liang, M. Hada, M. Ehara, K. Toyota, R. Fukuda, J. Hasegawa, M. Ishida, T. Nakajima, Y. Honda, O. Kitao, H. Nakai, T. Vreven, K. Throssell, J. A. Montgomery Jr., J. E. Peralta, F. Ogliaro, M. J. Bearpark, J. J. Heyd, E. N. Brothers, K. N. Kudin, V. N. Staroverov, T. A. Keith, R. Kobayashi, J. Normand, K. Raghavachari, A. P. Rendell, J. C. Burant, S. S. Iyengar, J. Tomasi, M. Cossi, J. M. Millam, M. Klene, C. Adamo, R. Cammi, J. W. Ochterski, R. L. Martin, K. Morokuma, O. Farkas, J. B. Foresman. Gaussian 16 Rev. B.01, **2016**.
[35]
E. Epifanovsky, A. T. B. Gilbert, X. Feng, J. Lee, Y. Mao, N. Mardirossian, P. Pokhilko, A. F. White, M. P. Coons, A. L. Dempwolff, Z. Gan, D. Hait, P. R. Horn, L. D. Jacobson, I. Kaliman, J. Kussmann, A. W. Lange, K. U. Lao, D. S. Levine, J. Liu, S. C. McKenzie, A. F. Morrison, K. D. Nanda, F. Plasser, D. R. Rehn, M. L. Vidal, Z.-Q. You, Y. Zhu, B. Alam, B. J. Albrecht, A. Aldossary, E. Alguire, J. H. Andersen, V. Athavale, D. Barton, K. Begam, A. Behn, N. Bellonzi, Y. A. Bernard, E. J. Berquist, H. G. A. Burton, A. Carreras, K. Carter-Fenk, R. Chakraborty, A. D. Chien, K. D. Closser, V. Cofer-Shabica, S. Dasgupta, M. de Wergifosse, J. Deng, M. Diedenhofen, H. Do, S. Ehlert, P.-T. Fang, S. Fatehi, Q. Feng, T. Friedhoff, J. Gayvert, Q. Ge, G. Gidofalvi, M. Goldey, J. Gomes, C. E. González-Espinoza, S. Gulania, A. O. Gunina, M. W. D. Hanson-Heine, P. H. P. Harbach, A. Hauser, M. F. Herbst, M. Hernández Vera, M. Hodecker, Z. C. Holden, S. Houck, X. Huang, K. Hui, B. C. Huynh, M. Ivanov, Á. Jász, H. Ji, H. Jiang, B. Kaduk, S. Kähler, K. Khistyaev, J. Kim, G. Kis, P. Klunzinger, Z. Koczor-Benda, J. H. Koh, D. Kosenkov, L. Koulias, T. Kowalczyk, C. M. Krauter, K. Kue, A. Kunitsa, T. Kus, I. Ladjánszki, A. Landau, K. V. Lawler, D. Lefrancois, S. Lehtola, R. R. Li, Y.-P. Li, J. Liang, M. Liebenthal, H.-H. Lin, Y.-S. Lin, F. Liu, K.-Y. Liu, M. Loipersberger, A. Luenser, A. Manjanath, P. Manohar, E. Mansoor, S. F. Manzer, S.-P. Mao, A. V. Marenich, T. Markovich, S. Mason, S. A. Maurer, P. F. McLaughlin, M. F. S. J. Menger, J.-M. Mewes, S. A. Mewes, P. Morgante, J. W. Mullinax, K. J. Oosterbaan, G. Paran, A. C. Paul, S. K. Paul, F. Pavošević, Z. Pei, S. Prager, E. I. Proynov, Á. Rák, E. Ramos-Cordoba, B. Rana, A. E. Rask, A. Rettig, R. M. Richard, F. Rob, E. Rossomme, T. Scheele, M. Scheurer, M. Schneider, N. Sergueev, S. M. Sharada, W. Skomorowski, D. W. Small, C. J. Stein, Y.-C. Su, E. J. Sundstrom, Z. Tao, J. Thirman, G. J. Tornai, T. Tsuchimochi, N. M. Tubman, S. P. Veccham, O. Vydrov, J. Wenzel, J. Witte, A. Yamada, K. Yao, S. Yeganeh, S. R. Yost, A. Zech, I. Y. Zhang, X. Zhang, Y. Zhang, D. Zuev, A. Aspuru-Guzik, A. T. Bell, N. A. Besley, K. B. Bravaya, B. R. Brooks, D. Casanova, J.-D. Chai, S. Coriani, C. J. Cramer, G. Cserey, A. E. DePrince, R. A. DiStasio, A. Dreuw, B. D. Dunietz, T. R. Furlani, W. A. Goddard, S. Hammes-Schiffer, T. Head-Gordon, W. J. Hehre, C.-P. Hsu, T.-C. Jagau, Y. Jung, A. Klamt, J. Kong, D. S. Lambrecht, W. Liang, N. J. Mayhall, C. W. McCurdy, J. B. Neaton, C. Ochsenfeld, J. A. Parkhill, R. Peverati, V. A. Rassolov, Y. Shao, L. V. Slipchenko, T. Stauch, R. P. Steele, J. E. Subotnik, A. J. W. Thom, A. Tkatchenko, D. G. Truhlar, T. Van Voorhis, T. A. Wesolowski, K. B. Whaley, H. L. Woodcock, P. M. Zimmerman, S. Faraji, P. M. W. Gill, M. Head-Gordon, J. M. Herbert, A. I. Krylov, *J. Chem. Phys.* **2021**, *155*, 084801.
